# A proposal for the withdrawal of inhaled corticosteroids in the clinical practice of chronic obstructive pulmonary disease

**DOI:** 10.1186/s12931-017-0682-y

**Published:** 2017-11-28

**Authors:** Marc Miravitlles, Borja G. Cosío, Aurelio Arnedillo, Myriam Calle, Bernardino Alcázar-Navarrete, Cruz González, Cristóbal Esteban, Juan Antonio Trigueros, José Miguel Rodríguez González-Moro, José Antonio Quintano Jiménez, Adolfo Baloira

**Affiliations:** 1 Pneumology Department, Hospital Universitari Vall d’Hebron, P. Vall d’Hebron 119-129, 08035 Barcelona, Spain; 2 CIBER de Enfermedades Respiratorias (CIBERES), Barcelona, Spain; 3 Department of Respiratory Medicine, Hospital Universitario Son Espases-IdISBa, Palma de Mallorca, Spain; 4 Pneumology, Allergy and Thoracic Surgery Department, Hospital Universitario Puerta del Mar, Cádiz, Spain; 5 Medicine Department, University of Cádiz, Cádiz, Spain; 6 Pulmonary Department, Instituto de Investigación Sanitaria del Hospital Clínico San Carlos (IdISSC), Hospital Clínico San Carlos, Madrid, Spain; 7 Departamento de Medicina, Facultad de Medicina, Universidad Complutense de Madrid, Madrid, Spain; 8 Respiratory Department, AIG de Medicina, Hospital de Alta Resolución de Loja, Agencia Sanitaria Hospital de Poniente, Loja, Granada Spain; 9 Department of Respiratory Medicine, Hospital Clínico Universitario and Instituto de Investigación Sanitaria (INCLIVA) Valencia, Valencia, Spain; 10 Pneumology Department of Hospital Galdakao-Usansolo, Biscay, Spain; 11 Red de Investigación en Servicios Sanitarios y Enfermedades Crónicas (REDISSEC), Bilbao, Spain; 12 Health Center Menasalbas, Autonomic Health Service, Toledo, Spain; 13 Pneumology Department, Hospital Universitary “Príncipe de Asturias”, Alcalá de Henares, Madrid, Spain; 14 Centro de Salud Lucena I, Lucena, Córdoba, Spain; 15 Servicio de Neumología, Complejo Hospitalario Universitario de Pontevedra, Pontevedra, Spain

**Keywords:** Algorithm, Chronic obstructive pulmonary disease, Exacerbations, Inhaled corticosteroids, Lung function

## Abstract

According to the current clinical practice guidelines for chronic obstructive pulmonary disease (COPD), the addition of inhaled corticosteroids (ICS) to long-acting β_2_ agonist therapy is recommended in patients with moderate-to-severe disease and an increased risk of exacerbations. However, ICS are largely overprescribed in clinical practice, and most patients are unlikely to benefit from long-term ICS therapy.

Evidence from recent randomized-controlled trials supports the hypothesis that ICS can be safely and effectively discontinued in patients with stable COPD and in whom ICS therapy may not be indicated, without detrimental effects on lung function, health status, or risk of exacerbations. This article summarizes the evidence supporting the discontinuation of ICS therapy, and proposes an algorithm for the implementation of ICS withdrawal in patients with COPD in clinical practice.

Given the increased risk of potentially serious adverse effects and complications with ICS therapy (including pneumonia), the use of ICS should be limited to the minority of patients in whom the treatment effects outweigh the risks.

## Background

Chronic obstructive pulmonary disease (COPD) is associated with high morbidity and mortality worldwide, [1] and continues to represent a significant public health challenge. According to the Global Initiative for Chronic Obstructive Lung Disease (GOLD) recommendations, long-acting bronchodilators are the mainstay of treatment in patients with COPD [2]. Similarly, all national guidelines for the management of COPD in Europe recommend bronchodilators as first-line therapy, regardless of clinical presentation or phenotype [3].

Anti-inflammatory therapy with inhaled corticosteroids (ICS) may be added to long-acting β_2_-agonists (LABAs) in patients with moderate-to-very severe COPD and exacerbations [2]. The ICS/LABA combination is more effective than either of the individual drugs alone for improving health status and lung function and reducing exacerbations in these patients, [4–6] and may also be used in the management of patients with asthma-COPD overlap (ACO) [2, 7, 8]. However, regular ICS therapy is associated with an increased risk of pneumonia, particularly in older patients (aged ≥55 years) and those with severe disease, as well an increased prevalence of oral candidiasis, skin bruising and hoarse voice [2, 9, 10].

According to the GOLD strategy, patients with COPD in group D (with a history of frequent exacerbations [≥2 in the past year], and a COPD Assessment Test [CAT] score ≥ 10 or modified Medical Research Council score ≥ 2) should receive first-line treatment with a LABA/long-acting muscarinic antagonist (LAMA) combination [2]. ICS/LABA combination therapy may be considered as first-line therapy in patients with suspected ACO or high blood eosinophil counts [2, 8]. Based on these combined risk criteria, real-life data indicate that only a minority of patients with COPD would be potential candidates for ICS therapy [11, 12]. However, ICS prescribing rates (either alone or as combination therapy) reportedly range from 42% to 86%, regardless of COPD severity and exacerbation risk [7, 13–18]. This high ICS usage significantly increases the risk of serious pneumonia and other respiratory infections [6, 19, 20].

In a 2015 Spanish consensus report, it was agreed that ICS therapy should be added to long-acting bronchodilators in patients with frequent exacerbations and in those with ACO phenotype, but should not be added to LABA therapy to improve lung function [21]. The expert panel also agreed that ICS withdrawal in patients with stable COPD was possible, although no consensus was reached on how, when and in whom to discontinue ICS [21]. Algorithms for the withdrawal of ICS in patients switched from GOLD D to C following the new GOLD classification [22] and for the stepwise ICS withdrawal in patients with COPD [23, 24] have previously been proposed; however, complex treatment algorithms are often difficult to adopt into clinical practice. To this end, simplified treatment algorithms for COPD management have been developed [25, 26].

As a follow-up to the previous Spanish consensus report on ICS use in COPD, experts in COPD management met with the aim of developing a simplified strategy for ICS withdrawal in patients with COPD. This article summarizes the evidence for the discontinuation of ICS therapy and presents a potential algorithm for the implementation of ICS withdrawal in clinical practice.

### Effects of ICS therapy in patients with COPD

#### Anti-inflammatory effects

ICSs are very effective as anti-inflammatory therapy in patients with asthma. ICS therapy works by recruiting histone deacetylase (HDAC) enzymes to inflammatory genes that are being actively transcribed; this suppresses the expression of inflammatory proteins and results in decreased airway inflammation [27]. Compared with asthma, ICS therapy is less effective when used in the management of COPD. The diminished anti-inflammatory effects of ICSs in COPD may be caused by decreased HDAC expression and enzyme activity, which has been observed in the alveolar macrophages of patients with COPD and is thought to be mediated by oxidative stress [28, 29].

Although the anti-inflammatory effects of ICS therapy are decreased in COPD compared with asthma, decreases in airway inflammation with ICS have been reported in patients with COPD. In a study of patients with moderate-to-severe COPD, inhaled fluticasone/salmeterol for 13 weeks led to significant reductions in absolute biopsy CD8^+^, CD4^+^, and CD45^+^ cell counts versus placebo, as well as sputum differential neutrophil counts and sputum eosinophils [30]. The Groningen Leiden Universities Corticosteroids in Obstructive Lung Disease (GLUCOLD)-1 study has also shown significant reductions in sputum neutrophils, macrophages, and lymphocyte counts with fluticasone therapy over 30 months in patients with moderate-to-severe COPD, while withdrawal of fluticasone after 6 months led to increased bronchial CD3^+^, mast, and plasma cell counts [31]. In a long-term observational follow-up study (GLUCOLD-2), patients who discontinued fluticasone after 30 months of treatment experienced an increase in sputum inflammatory cells, as well as bronchial T-lymphocytes and mast cells [32]. The GLUCOLD-2 study also demonstrated a significant correlation between increased sputum macrophage counts and an accelerated rate of decline in lung function [32]. These studies suggest that at least some patients with COPD, and in particular those with significant airway inflammation, may benefit from ICS therapy. However, there is also evidence that fluticasone may not affect airway inflammation. For example, a study of Japanese patients with COPD showed no significant changes from baseline in sputum neutrophils or other inflammatory markers with salmeterol/fluticasone for 12 weeks when compared with placebo [33].

Eosinophilic airway inflammation, which is commonly found in patients with asthma, also occurs in some patients with COPD [34]. High blood and/or sputum eosinophil counts are often associated with an increased risk of COPD exacerbations, [35, 36] and may be a marker of response to ICS therapy [37–39].

#### Clinical effects

Early randomized-controlled trials (RCTs) have demonstrated the clinical benefits of inhaled fluticasone propionate in patients with moderate-to-severe COPD [40, 41]. In these trials, fluticasone was associated with significantly lower rates of moderate or severe exacerbations compared with placebo over 6 months, [41] or 3 years [40].

Subsequent studies have shown that ICS/LABA combination therapy is more effective than ICS alone, particularly with regard to reducing exacerbations, [5] but has no effect on long-term survival. In the Towards a Revolution in COPD Health (TORCH) trial, fluticasone propionate/salmeterol was associated with a slight reduction in mortality risk compared with placebo, but this reduction was not statistically significant [42]. The mortality risk also did not significantly differ between fluticasone/salmeterol and salmeterol alone, but was significantly lower with the combination therapy than fluticasone alone (*p* = 0.007) [42]. A subsequent analysis of data from the TORCH study suggested that any mortality benefit from fluticasone/salmeterol versus placebo was more likely attributable to the salmeterol component [43]. In the Study to Understand Mortality and Morbidity (SUMMIT) in patients with moderate COPD and increased cardiovascular risk, fluticasone furoate/vilanterol had no significant effect on all-cause mortality or cardiovascular outcomes compared with placebo [44].

The beneficial effects of ICS therapy in preventing or reducing exacerbations appear to be limited to patients with eosinophilic inflammation. In a randomized study of patients with COPD, a 62% mean reduction in the frequency of severe exacerbations was observed when COPD management was aimed at minimizing sputum eosinophil levels compared with conventional guideline-based management [45]. In a post hoc analysis of two randomized, double blind trials, the combination of vilanterol/fluticasone furoate was shown to reduce COPD exacerbations by 29% in patients with eosinophil counts of ≥2% and by 10% in patients with eosinophil counts of <2% compared with vilanterol alone [46]. In a post hoc analysis of the Foster 48-Week Trial to Reduce Exacerbations in COPD (FORWARD), the beneficial effects of beclomethasone dipropionate/formoterol fumarate (ICS/LABA) versus formoterol fumarate alone with regard to reductions in exacerbations were most evident in patients with peripheral blood eosinophilia (≥279.8 cells/μL) [39]. Similarly, a pooled analysis of two 12-month RCTs showed that patients with >2.4% blood eosinophils potentially achieve the greatest reductions in exacerbations with ICS/LABA therapy versus LABA alone [38]. An analysis of the Inhaled Steroids in Obstructive Lung Disease in Europe (ISOLDE) trial indicated that a baseline blood eosinophil count ≥2% was associated with a significantly reduced rate of FEV_1_ decline with fluticasone propionate versus placebo (*p* = 0.003), while there was no difference in FEV_1_ decline among those with <2% eosinophils [37].

However, when comparing the efficacy of ICS with that of long-acting bronchodilators the results are usually favorable to the latter. LAMA alone has been shown to be as effective as ICS/LABA therapy with regard to improvements in lung function and preventing exacerbations in patients with COPD. In the Investigating New Standards for Prophylaxis In Reducing Exacerbations (INSPIRE) study of patients with severe or very severe COPD and a history of exacerbations, the annual rate of exacerbations showed no difference between fluticasone propionate/salmeterol and tiotropium bromide alone, although the incidence of pneumonia was significantly higher with fluticasone propionate/salmeterol (*p* = 0.008) [47].

The LABA/LAMA combination indacaterol/glycopyrronium was associated with significantly greater improvements in lung function over 26 weeks compared with fluticasone/salmeterol (*p* < 0.001) in the Efficacy and safety of once-daily indacaterol/glycopyrronium compared with twice-daily salmeterol–fluticasone in patients with chronic obstructive pulmonary disease (ILLUMINATE) trial, [48] and significantly reduced the rate of moderate or severe exacerbations with a lower rate of pneumonia in A 26-week treatment randomized, double-blind, double dummy, parallel-group study to assess the efficacy and safety of indacaterol/glycopyrronium (LANTERN) trial [49]. In the Effect of indacaterol/glycopyronium vs fluticasone/salmeterol on COPD exacerbations (FLAME) trial, the annual rate of COPD exacerbations was also found to be significantly (11%) lower with indacaterol/glycopyrronium than fluticasone/salmeterol over 12 months (*p* = 0.003) in patients with a previous history of at least one exacerbation. It is important to indicate that patients with a history of asthma and/or blood eosinophil counts >600 cells/μL were excluded form FLAME [50].

#### Adverse effects of inhaled corticosteroids

Accumulated evidence from clinical trials indicates that ICS therapy is associated with a high risk of pneumonia, oral candidiasis, hoarse voice, and skin bruising [2, 4, 5, 9, 10]. The risk of pneumonia is increased in older patients (aged ≥55 years), current smokers, and patients with a history of exacerbations or pneumonia, a body mass index <25 kg/m^2^, dyspnea, and/or severe airflow limitation [2, 9]. In a meta-analysis of RCTs in patients with COPD, inhaled fluticasone or budesonide was associated with a significant (57%) increase in the risk of pneumonia (*p* < 0.0001) [51]. However, an observational cohort study of patients with COPD found that the rates of pneumonia and hospitalization were significantly higher with fluticasone/salmeterol than budesonide/formoterol (*p* < 0.001), which suggests there are intra-drug class differences in the risks of pneumonia with ICS/LABA combination therapies [52].

Long-term ICS exposure may also be associated with an increased risk of bone fractures in patients with COPD [53]. A meta-analysis of RCTs and observational studies indicated a significant (27%) increase in the risk of fractures with fluticasone or budesonide therapy (*p* = 0.04) [53]. Osteoporosis and COPD are also strongly correlated due to common lifestyle risk factors (eg physical inactivity, poor diet, and smoking), COPD-associated inflammation, and vitamin D deficiency [54].

Other adverse effects associated with the use of ICS include an increased risk of new-onset diabetes or diabetes progression, [55] cataracts, [56] and tuberculosis [57]. In a database cohort study of patients with respiratory disease, ICS therapy was associated with a 34% increase both in the risk of new-onset diabetes and in the risk of diabetes progression [55].

### Evidence supporting ICS withdrawal in patients with COPD

The excessive and inappropriate use of ICS in COPD together with the increased risk of adverse effects associated with its use makes it necessary to discontinue this treatment in patients in which the risks overweight the possible benefits. Withdrawal of ICS has been analyzed in several studies.

In patients with COPD, ICS withdrawal was associated with several clinical disadvantages in early studies (Table 1). As part of the ISOLDE trial, [40] an observational study compared patients who were receiving ICS at study entry with those who were not on ICS [58]. In this 8-week study, ICS withdrawal was associated with more exacerbations, indicating that patients should be monitored carefully following abrupt ICS discontinuation [58]. In the Effect of discontinuation of inhaled corticosteroids in patients with chronic obstructive pulmonary disease (COPE) study, withdrawal of inhaled fluticasone led to a higher risk of exacerbation and a significant decline in quality of life [59]. Likewise, discontinuation of fluticasone was associated with a decline in lung function and an increase in dyspnea and mild exacerbations in the COPD and Seretide: a Multicenter Intervention and Characterization (COSMIC) trial, [60] and a worsening of symptoms and increased risk of exacerbations in the Withdrawal of inhaled corticosteroids in people with COPD in primary care (WISP) trial [61].

**Table 1:** Studies of inhaled corticosteroid (ICS) withdrawal in patients with chronic obstructive pulmonary disease

Author, year (study name)	Study design; duration	Study population	Treatment	Main findings
Jarad, 1999 [58]	MC, observational study; 8 weeks	COPD, post-BD FEV_1_ < 70% predicted (*n* = 272)	ICS withdrawal vs no previous ICS therapy	Increased risk of exacerbation after ICS withdrawal
van der Valk, 2002 (COPE) [59]	R, DB, SC, PC trial;4-month ICS run-in,6-month active treatment	Moderate-to-severe COPD, pre-BD FEV_1_ 25–80% predicted, no exacerbations in past month (*n* = 244)	FP/IB vs PBO+ IB	Higher risk of exacerbation and deterioration in QoL after ICS withdrawal
Wouters, 2005 (COSMIC) [60]	R, DB, PG, MC trial; 3-month ICS/LABA run-in, 1-year active treatment	Moderate-to-severe COPD, pre-BD FEV_1_ 30–70% predicted, ≥2 exacerbations in past year (*n* = 373)	FP/Sal vs Sal alone	Deterioration of symptoms and lung function after ICS withdrawal; no difference in the rate of exacerbations
Choudhury, 2007 (WISP) [61]	R, DB, PC trial; 2 week run-in on usual ICS; 1-year treatment	Primary care, moderate-to-severe COPD, post-BD FEV_1_ < 80% predicted, no exacerbations in past month(*n* = 260)	FP vs PBO	Higher risk of exacerbations and symptom deterioration after ICS withdrawal; no differences in lung function or QoL
Magnussen, 2014 (WISDOM) [63]	R, DB, PG, MC trial; 6-week ICS/LABA/LAMA run-in; 12-month active treatment	Severe-to-very severe COPD, FEV_1_ < 50%, ≥1 exacerbation in past year (*n* = 2485)	FP/Sal/Tio vs Sal/Tio + stepwise FP withdrawal	No differences in symptoms or exacerbations rate; significantly greater decrease in lung function with complete ICS withdrawal
Rossi, 2014 (INSTEAD) [65]	R, DB, DD, PG trial; 2-week ICS/LABA run-in; 26-week active treatment	Moderate COPD, GOLD stage II, no exacerbations in the past year (*n* = 581)	Switching from FP/Sal to Ind vs continuing FP/Sal	No differences in exacerbations, symptoms, lung function, QoL
Rossi, 2014 (OPTIMO) [66]	Prospective, real-life study; 6-month observation period	Mild-to-moderate COPD, on ICS + LABA for 1 year, FEV_1_ > 50%, <2 exacerbations in past year (*n* = 914)	ICS/LABA vs mainly LABD (91% LABA ± LAMA; 9% SABD ± theophylline)	ICS withdrawal not associated with worsening of exacerbation rates, lung function or symptoms
Suissa, 2015 [68]	Population-based, observational study; 4.9 years of follow-up	COPD treated with ICS (*n* = 103,386)	ICS withdrawal	Significant 37% reduction in serious pneumonia after ICS withdrawal; greatest reduction after FP withdrawal
Vogelmeier, 2013 (ILLUMINATE) [48]	R, DB, DD, PG, MC trial; 26 weeks	Moderate-to-severe COPD, GOLD stages II–III, no exacerbations in past year (*n* = 523)	Ind/Gly vs FP/Sal	Significantly improved lung function with Ind/Gly
Zhong, 2015 (LANTERN) [49]	R, DB, DD, PG, MC trial; 26 weeks	Moderate-to-severe COPD, GOLD stage II–III, ≤1 exacerbation in past year (*n* = 744)	Ind/Gly vs FP/Sal	Significantly reduced exacerbations and improved lung function with Ind/Gly; incidence of pneumonia 3-fold lower with Ind/Gly
Wedzicha, 2016 (FLAME) [50]	R, DB, DD, PG, MC trial; 4-week Tio run-in; 52-week active treatment	Moderate-to-severe COPD, post-BD FEV_1_ 25–60% predicted, ≥1 exacerbation in past year (*n* = 3362)	Ind/Gly vs FP/Sal	Lower annual rate of exacerbation and longer time to first exacerbation with Ind/Gly; incidence of pneumonia higher with FP/Sal
Vogelmeier, 2017 (CRYSTAL) [69]	R, OL, MC trial; 12 weeks	Moderate COPD, ≤1 exacerbation in past year, on ICS/LABA, LABA, or LAMA therapy (*n* = 4389)	Switching to Gly or Ind/Gly vs continuing baseline therapy	Improved lung function and dyspnea with Ind/Gly vs ongoing ICS/LABA, LABA, or LAMA therapy

*BD* bronchodilator, *COPD* chronic obstructive pulmonary disease, *DB* double-blind, *DD* double-dummy, *FEV*_*1*_ forced expiratory volume in 1 s, *FP* fluticasone propionate, *Gly* glycopyrronium, *IB* ipratropium bromide, *ICS* inhaled corticosteroid, *Ind* indacterol, *LABA* long-acting β-agonist, *LAMA* long-acting muscarinic antagonist, *LABD* long-acting bronchodilator, *MC* multicenter, *PBO* placebo, *PC* placebo-controlled, *PDE* phosphodiesterase, *PG* parallel-group, *Pred* prednisolone, *QoL* quality of life, *R* randomized, *Sal* salmeterol, *SABD* short-acting bronchodilator, *Tio* tiotropium

However, a meta-analysis of the WISP, COPE, and COSMIC trials indicated that ICS withdrawal was not associated with a significant increase in the risk of exacerbations [62]. The definition of exacerbation differed between these early studies and the use of other medication was not reported [62]. A more recent and robust meta-analysis concluded that ICS discontinuation did not significantly increased the overall rate of COPD exacerbations, although an increased risk of severe exacerbations was detected [63]. The increased risk of all types of exacerbations following ICS withdrawal observed in early studies was most likely due to a lack of alternative COPD medications.

Evidence from more recent RCTs and real-life studies supports the hypothesis that the ICS therapy can be safely withdrawn in patients with stable COPD (Table 1). In the Withdrawal of Inhaled Steroids During Optimized Bronchodilator Management (WISDOM) trial, in which the safety of gradual ICS withdrawal in patients previously on ICS/LABA/LAMA triple therapy was evaluated, there was no significant difference in the risk of moderate or severe COPD exacerbation over 12 months after ICS withdrawal compared with ongoing triple therapy [64]. Although a significantly greater decrease in FEV_1_ was observed at 18 weeks after ICS withdrawal (mean difference 38 mL vs continued ICS; *p* < 0.001), [63] this was less than half of what is considered as the minimum clinically important difference. A post hoc analysis of WISDOM showed that patients who stopped ICS experienced a similar disease course with regard to lung function to those who continued ICS therapy [64]. Although deterioration of lung function was observed after ICS withdrawal, these changes were small, not progressive and not predictive of clinically important changes over the duration of study follow-up [65].

By contrast, in the Indacaterol: Switching Non-exacerbating Patients with Moderate COPD from Salmeterol/Fluticasone to Indacaterol (INSTEAD) trial, there were no clinically relevant reductions in lung function or differences in dyspnea or health status over 26 weeks among patients who switched to indacaterol compared with those who continued fluticasone/salmeterol therapy [66]. The annual rate of mild, moderate, or severe COPD exacerbations also showed no significant difference between the groups. The INSTEAD trial concluded that patients with moderate airflow limitation and no history of exacerbations can be switched from fluticasone/salmeterol to indacaterol monotherapy without loss in treatment efficacy [66].

In the Real-life study on the appropriateness of treatment in moderate COPD patients (OPTIMO), the risk of exacerbations did not significantly increase over 6 months after ICS withdrawal compared with continued ICS/bronchodilator therapy [67]. There was also no evidence of deterioration in COPD symptoms or lung function over 6 months following ICS withdrawal [67]. Consistent with these findings, a subgroup analysis of patients on ICS prior to study entry in the Outpatient care with long-acting bronchodilators: COPD registry in Germany (DACCORD) showed that ICS withdrawal was not associated with an increase in the risk of exacerbations or an increased risk of health status deterioration compared with continued ICS therapy [68]. Indeed, in the second year of follow-up in DACCORD, the annual rate of exacerbations was lower among patients who underwent ICS withdrawal than in those who continued ICS therapy [68].

ICS withdrawal may also provide clinical benefits in patients by reducing the risk of adverse effects, particularly pneumonia. In a population-based cohort study, ICS withdrawal in patients with COPD was associated with a 37% decrease in the rate of serious pneumonia over 3 years, with a 20% risk reduction over the first month [69]. Of note, patients who discontinued fluticasone showed greater reductions in severe pneumonia risk than those who discontinued budesonide [69]. These findings indicate that the beneficial effects of ICS withdrawal may occur soon after treatment discontinuation.

In a subanalysis of exacerbation rates by previous therapy in the FLAME trial, among patients who had previously received ICS therapy (56% of patients), those who were randomized to LABA/LAMA had a significantly lower risk of exacerbations than those who continued ICS/LABA therapy (risk ratio 0.88; 95% confidence interval 0.80–0.97) [50]. Additionally, the effect of glyCopyrronium or indacateRol maleate and glYcopyrronium bromide fixed-dose combination on SympToms and heALth status in patients with moderate COPD (CRYSTAL) study of symptomatic patients with moderate COPD previously on ICS/LABA, LABA or LAMA therapy showed that switching to indacaterol/glycopyrronium was associated with significant improvements in trough FEV_1_ over 12 weeks [70].

In a subanalysis of the WISDOM study, patients with severe or very severe COPD and a history of exacerbations who had blood eosinophil counts of ≥4% (≥300 cells/μL) were at an increased risk of exacerbations following ICS withdrawal compared with those with lower eosinophil counts [71]. In fact, the risk of exacerbation after withdrawal was significantly increased in patients who suffered 2 or more exacerbations the previous year and had blood eosinophil counts >300 cells/μL. In addition, these patients experienced a mean decrease of 109 mL in FEV1 compared with the mean decrease of 43 mL for the whole population [72]. This suggests that ICS withdrawal may have deleterious effects in a subpopulation of patients with higher blood eosinophil counts and frequent exacerbations.

### Proposed algorithm for ICS withdrawal in patients with COPD

**Fig. 1:**
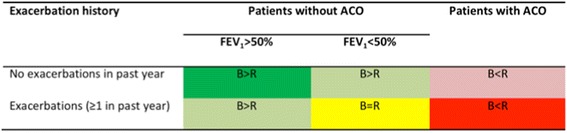
Patient categories based on exacerbation history, airflow limitation and the risks of inhaled corticosteroid withdrawal. ACO, asthma-chronic obstructive pulmonary disease overlap; B, benefits of ICS withdrawal; FEV1, forced expiratory volume in 1 s; ICS, inhaled corticosteroid; R, risks associated with ICS withdrawal; dark green = ICS should be discontinued; red = ICS should not be withdrawn; pale green, pale red, or yellow = ICS withdrawal should be carefully evaluated on a case-by-case basis, taking into account the risk of ICS-associated adverse effects

**Fig. 2:**
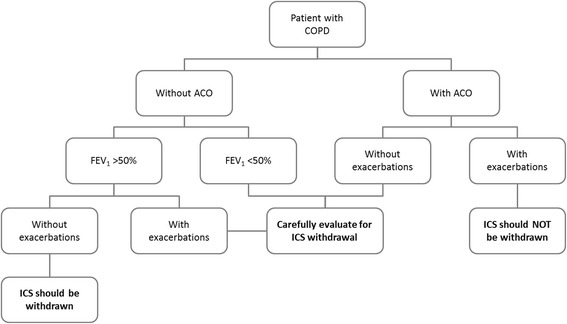
Proposed algorithm for ICS withdrawal in patients with COPD. ACO, asthma-COPD overlap; COPD, chronic obstructive pulmonary disease; FEV_1_, forced expiratory volume in 1 s; ICS, inhaled corticosteroid. ACO is diagnosed according to the Spanish consensus as COPD plus asthma diagnosis or COPD and blood eosinophil counts >300 cells/μL and/or a post-bronchodilator response of >400 mL and 15% in FEV_1_

The proposal for managing ICS withdrawal in patients with COPD takes three clinical parameters into account: a) whether or not there is a history of previous exacerbations; b) whether FEV_1_ is more or less than 50%; and c) whether the patient has criteria for ACO (which in the Spanish guidelines includes patients with COPD and blood eosinophil counts >300 cells/μL and/or a post-bronchodilator response of >400 mL and 15% in FEV_1_) [73]. Based on these clinical parameters, patients with COPD may be classified into three categories: (i) patients in whom the risks associated with ICS withdrawal exceed the benefits; (ii) those in whom the benefits of ICS withdrawal are greater than the risks; and (iii) those in whom the risks and benefits of ICS are balanced. A summary of this patient classification and how lung function and exacerbation history affect the benefits and risks of ICS withdrawal is shown in Fig. 1. Taking these categories into consideration, the decision to withdraw or continue ICS is based on the following:

1. In patients with COPD, FEV_1_ > 50% and no previous exacerbations, the benefits of ICS withdrawal exceed risks and ICS must be withdrawn.
2. In patients with ACO and exacerbations in the previous year, the risks associated with ICS withdrawal exceed the benefits and ICS should not be withdrawn.
3. Patients with FEV_1_ > 50% and exacerbations in the previous year and patients with FEV_1_ < 50% without exacerbations have an intermediate level of risk associated with ICS withdrawal. The risk of exacerbations after ICS discontinuation is low, but dual bronchodilator therapy should be maintained to make sure that the risk of exacerbations does not increase.
4. Patients with FEV_1_ < 50% and exacerbations in the previous year, together with patients with ACO without exacerbations, may have an increased risk of exacerbations after ICS withdrawal. Discontinuation should be considered only in patients with a significant risk of serious ICS-related adverse effects. In these patients, ICS withdrawal may still be possible provided that dual bronchodilator therapy is maintained, but close follow-up is essential.
  An overview of the proposed algorithm is shown in Fig. 2.

#### Benefits of ICS withdrawal exceed risks

In patients with FEV_1_ > 50% and no exacerbations in the previous year, the benefits of ICS withdrawal exceed the risks and ICS therapy should be discontinued. Evidence supporting ICS withdrawal in these patients is provided by the OPTIMO, [67] INSTEAD, [66] and DACCORD [74] studies, in which patients with mild or moderate COPD had no changes in exacerbations, lung function, or health status following ICS withdrawal.

Studies have also indicated that dual bronchodilator therapy with a LABA/LAMA combination may be used as an alternative to ICS/LABA therapy in patients with moderate-to-severe COPD and a low risk of exacerbations. In this group of patients, LABA/LAMA therapy (indacaterol/glycopyrronium) was associated with improved lung function and lower rates of exacerbation and pneumonia compared with ICS/LABA therapy (fluticasone/salmeterol) in the ILLUMINATE, [48] LANTERN, [49] and CRYSTAL [70] studies. Therefore, patients with an FEV_1_ > 50% and no exacerbations in the previous year are candidates for ICS withdrawal, provided that dual bronchodilator therapy is continued [75].

#### Risks associated with ICS withdrawal exceed benefits

In patients with ACO and a history of exacerbations in the previous year, the ICS withdrawal risks are greater than the benefits and ICS should not be discontinued. ACO is recognized as a distinct COPD phenotype, and relevant clinical practice guidelines provide criteria to enable clinicians to identify patients with COPD who have an asthma component [8, 76]. The prevalence of ACO among patients with COPD is thought to be up to 20%, [20, 77–83] although prevalence is difficult to stimate as there is no internationally accepted definition of ACO [84, 85].

A simplified algorithm for the identification of ACO was proposed following a consensus between the Spanish COPD and asthma guidelines [73]. In this algorithm, the first criterion to be met is a diagnosis of COPD (ie aged ≥35 years, ≥10 pack-year smoker or ex-smoker, and post-bronchodilator FEV_1_/FVC <0.7), after which the diagnostic criteria for asthma are assessed. If the diagnostic criteria for both COPD and asthma are met, a diagnosis of ACO is confirmed; patients who do not meet all of the asthma diagnostic criteria, but have a bronchodilator response of ≥400 mL and 15% in FEV_1_ and/or eosinophilia of ≥300 cells/μL are also classified as having ACO [73, 86]. Patients with non-completely reversible airflow obstruction but who smoked less than 10 pack-years should be considered and treated like asthmatics.

A post hoc analysis of the WISDOM trial identified a small subgroup of patients with COPD with a significantly increased risk of exacerbations after ICS withdrawal; these patients had a history of frequent exacerbations (≥2 per year) and high baseline eosinophil counts (≥300 or ≥400 cells/μL; defined as ACO according to Spanish guidelines), although a history of frequent exacerbations alone was not predictive of ICS responsiveness in the overall study population [72]. As these patients represented a very small proportion of the total patient population in the WISDOM study, this category is expected to consist of a minority of the patients with COPD.

#### Intermediate ICS withdrawal risk: benefit ratio

Patients with FEV_1_ > 50% and exacerbations in the previous year and patients with FEV_1_ < 50% without exacerbations have an intermediate risk associated with ICS withdrawal. Patients should be individually assessed to determine whether or not ICS therapy should be discontinued, with the risk of ICS-related adverse effects being taken into consideration. This recommendation is supported by evidence from the WISDOM [64] and FLAME [50] studies, in which patients with moderate, severe, or very severe COPD and a history of exacerbations showed no increase in the annual rate of exacerbations after discontinuation of ICS and ongoing dual bronchodilator therapy. In the FLAME study, the rate of pneumonia was lower with LABA/LAMA than ICS/LABA therapy, [50] and a post hoc analysis indicated that the lower rate of exacerbations with LABA/LAMA was independent of blood eosinophil levels [87]. Although the risk of exacerbations may be increased in patients with FEV_1_ < 50% and exacerbations in the previous year, as well as patients with ACO without exacerbations, ICS withdrawal needs to be considered in these patients, particularly those with an increased risk of serious ICS-related adverse effects. Following ICS withdrawal, these patients should be maintained on dual bronchodilator therapy and closely followed for exacerbations.

#### Method of ICS withdrawal

Based on clinical evidence, ICS therapy may be discontinued abruptly, rather than with gradual dose reduction. In the INSTEAD, [66] FLAME, [50] OPTIMO, [67] CRYSTAL, [70] and DACCORD [74] studies, ICS therapy was withdrawn abruptly with no apparent increase in exacerbations or loss of lung function. WISDOM was the only study in which the ICS dose was decreased in a stepwise fashion [64]. Although ICS withdrawal was associated with a significant decrease in FEV_1_ in this study, the decline in lung function was only observed after complete ICS discontinuation at 18 weeks and was not progressive [64, 65]. This suggests that the effect of ICS withdrawal on lung function (if any) only occurs after complete discontinuation, and there is no need to taper the dose in most patients. It should be noted that the LABA used in this study was salmeterol, which has a low intrinsic efficacy and a well-known tolerance in terms of reduction of effect over time [88]. Particular care should be exercised in high-risk patients with frequent exacerbations or poor lung function receiving high doses of ICS, and routine follow-up of patients after ICS withdrawal is recommended.

## Conclusions

Physicians should carefully evaluate patients and provide individualized COPD treatment, especially when considering the initiation of ICS therapy or the safe discontinuation of ICS in patients on long-term therapy. Given the limited efficacy and the potentially serious adverse effects and complications of long-term ICS therapy, the use of ICS should be limited to the minority of patients with COPD in whom the treatment effects outweigh the risks, and patients for whom safe ICS withdrawal can be achieved should be identified.

## Abbreviations

ACO: Asthma-COPD overlap
CAT: COPD Assessment Test
COPD: Chronic obstructive pulmonary disease
COPE: Effect of discontinuation of inhaled corticosteroids in patients with chronic obstructive pulmonary disease
COSMIC: COPD and Seretide: a Multicenter Intervention and Characterization
CRYSTAL: Effect of glyCopyrronium or indacateRol maleate and glYcopyrronium bromide fixed-dose combination on SympToms and heALth status in patients with moderate COPD
DACCORD: Outpatient care with long-acting bronchodilators: COPD registry in Germany
FLAME: Effect of indacaterol glycopyronium vs fluticasone salmeterol on COPD exacerbations
FORWARD: Foster 48-week trial to reduce exacerbations in COPD; GOLD, Global Initiative for Chronic Obstructive Lung Disease
GLUCOLD: Groningen Leiden Universities Corticosteroids in Obstructive Lung Disease
HDAC: Histone deacetylase
ICS: Inhaled corticosteroids
ILLUMINATE: Efficacy and safety of once-daily QVA149 compared with twice-daily salmeterol–fluticasone in patients with chronic obstructive pulmonary disease
INSPIRE: Investigating New Standards for Prophylaxis In Reducing Exacerbations
INSTEAD: Indacaterol: Switching non-exacerbating patients with moderate COPD from salmeterol/fluticasone to indacaterol
ISOLDE: Inhaled Steroids in Obstructive Lung Disease in Europe
LABA: Long-acting β2-agonists; LAMA, long-acting muscarinic antagonist
LANTERN: A 26-week treatment randomized, double-blind, double dummy, parallel-group study to assess the efficacy and safety of QVA149
OPTIMO: Real-life study on the appropriateness of treatment in moderate COPD patients
RCT: Randomized-controlled trials
SUMMIT: Study to understand mortality and morbidity
TORCH: Towards a revolution in COPD health
WISDOM: Withdrawal of inhaled steroids during optimized bronchodilator management
WISP: Withdrawal of inhaled corticosteroids in people with COPD in primary care
